# Immune biomarkers in thymic epithelial tumors: expression patterns, prognostic value and comparison of diagnostic tests for PD-L1

**DOI:** 10.1186/s40364-019-0177-8

**Published:** 2019-12-04

**Authors:** Isabelle Rouquette, Estelle Taranchon-Clermont, Julia Gilhodes, Maria-Virginia Bluthgen, Romain Perallon, Lara Chalabreysse, Anne De Muret, Veronique Hofman, Alexander Marx, Marie Parrens, Veronique Secq, Vincent Thomas de Montpreville, Françoise Galateau-Salle, Pierre Brousset, Julie Milia, Nicolas Girard, Benjamin Besse, Thierry Jo Molina, Julien Mazières

**Affiliations:** 1 IUCT-Oncopole, 1 Avenue Irène Joliot Curie, 31059 Toulouse, France; 2 Gustave Roussy, 114 rue E Vaillant, 94805 Villejuif, France; 3 HCL, Hôpital Louis Pradel, 28 Avenue du Doyen Jean Lépine, 69500 Bron, France; 4 Hôpital Trousseau, Avenue de la République, 37170 Chambray-lès-Tours, France; 5 Hôpital Pasteur CHU, 30 voie Romaine, 06000 Nice, France; 6 Institut de Pathologie, Universitaetsmedizin Mannheim, Heidelberg University, D-68167 Mannheim, Germany; 7 Hôpital Haut-Levêque CHU, Avenue de Magellan, 33604 Pessac, France; 8 APHM Hôpital Nord, Chemin des Bourrely, 13915 Marseille, France; 9 Centre Chirurgical Marie-Lannelongue, 133 Avenue de la Résistance, 92350 Le Plessis- Robinson, France; 10 Centre Léon Bérard, 28 rue Laennec -, 69008 Lyon, France; 11 Hôpital Larrey, Centre Hospitalier Universitaire de Toulouse, 24 Chemin de Pouvourville, 31059 Toulouse, France; 12 Institut du Thorax Curie Montsouris, Institut Curie, 26, Rue d’Ulm, 75005 Paris, France; 13 Gustave Roussy, 114 rue E Vaillant, 94805 Villejuif, France; 14 Paris-Sud university, Orsay, France; 15 Hôpital Necker Enfants Malades, AP-HP, Université de Paris, 149 Rue de Sèvres, 75015 Paris, France

**Keywords:** PD-L1, Immunotherapy, Thymic carcinoma, B3 thymoma

## Abstract

**Background:**

Immunotherapy is currently under investigation in B3 Thymoma (TB3) and Thymic Carcinoma (TC). PD-L1 expression has been evaluated on a limited number of patients with selected antibodies. We aimed to analyze cohort of TB3 and TC with a panel of antibodies to assess the prevalence of PD-L1 expression, its prognostic value and to set up a reproducible test.

**Methods:**

We retrospectively studied 103 patients samples of FFPE histologically confirmed TB3 (*n* = 53) and TC (*n* = 50) by expert pathologists within the RYTHMIC national network. We compared PD-L1, PD1, CD8 and PD-L2 expression and performed correlation with tumor types and patients outcomes. Four PD-L1 antibodies were tested, three of them validated as companion tests in lung cancer, one tested on two automates on whole section of tumors. We evaluated the percentage and intensity of both epithelial and immune stained cells.

**Results:**

TB3 epithelial cells had a higher and more diffuse expression of PD-L1 than TC regardless the antibodies tested (*p* < 0.0001). Three out of four antibodies targeting PD-L1 tested on the DAKO autostainer gave similar staining. Concordance between antibodies was lower for PD-L1 staining on immune cells with no significant difference between TB3 and TC except on E1L3N antibody. PD-L2 antibody stained no tumor epithelial cells. High PD-L1 expression was correlated with a better overall survival for TB3 and was not correlated with tumor staging.

**Conclusion:**

Frequent PD-L1 expression, particularly in TB3, paves the way for immunotherapy in TET (Thymic Epithelial Tumor). Otherwise, we have set up three reproducible LDT (laboratory-developed test) for four PD-L1 antibodies.

## Introduction

Thymic epithelial tumors are rare. They represent a wide range of anatomical, histological, clinical, and molecular malignant entities, which may be aggressive and difficult to treat [1]. Most of them are surgically removed, either as a primary intervention for well-circumscribed tumors or after a neoadjuvant treatment. Into the WHO classification, there are five main subtypes (A, AB, B1, B2, and B3) which can be broadly divided into thymomas containing a majority of epithelial cells and thymomas composed of neoplastic epithelial cells mixed with variable abundance of immature T-cells. Type B3 thymoma (TB3) has a poor prognosis due to an often late stage diagnosis. Thymic carcinomas (TC) are set apart from thymoma as a diverse group of tumors with overt, often high grade, malignant behavior [2]. These two latter types of tumors are not always eligible for a surgical treatment due to their invasive properties. Chemotherapy and radiotherapy are thus often recommended with inconstant results [3]. No targeted therapy validated in lung cancer has been shown to be efficient for these tumors due to the lack of known oncogenic molecular alterations. Anti-angiogenic agents [4], cKIT [5] and mTOR inhibitors [6] have been tested in limited series of stage IV diseases.

The thymus is a crucial organ for the development of the immune system, especially for the selection of T-cells with appropriate self-tolerance. Although the physiopathology is not elucidated, auto-immune diseases are frequently associated with B1 and B2 subtypes, in particular myasthenia gravis. Immunotherapy may be a promising option for the treatment of advanced refractory TET (Thymic Epithelial Tumor) that are rarely associated with auto-immune diseases. Meanwhile, immunotherapy has recently entered the arsenal of therapeutic strategies in lung cancer [7]. The efficacy of immunotherapy is known to be correlated with the level of PD-L1 expression [8, 9]. Many different clones of PD-L1 antibodies have been tested in different tumors, in academic studies or clinical trials, as on different immunohistochemistry automates. In thymic tumors, early clinical trials have reported promising efficacy of PD1 inhibitors. In a phase 2 study, PD-L1 immunohistochemistry data were available for 37 thymic carcinomas. Positive staining (Dako 22C3) for PD-L1 in at least 50% tumors indicating high PD-L1 expression, was found in ten (25%) patients, six of whom had presented a partial or complete response [10]. The expression of PD-L1 in thymoma has been lately reported reaching from 23 to 70% according to tumor subtypes [11–13]. Nevertheless, immunohistochemistry was performed on tumor microarrays with single antibodies being rarely used in the development of current checkpoint inhibitors. The main objective of our work was to compare four major existing PD-L1 antibodies, three of them validated as companion tests in lung cancer in a national cohort of both TB3 and TC. The secondary objective was to compare PD-L1 expression to PD1, CD8 and PD-L2 expression and to correlate results with tumor types and patient’s outcomes.

## Materials and methods

### Patients

A total of 103 samples of FFPE histologically confirmed TB3 (*n* = 53) and TC (*n* = 50) from the RYTHMIC National Network have been analyzed [14]. Twenty samples were biopsies and 83 were surgically resected tumors. For each sample, the diagnosis was centrally reviewed by a national panel of pathologists according to the latest 2015 WHO classification [15]. Clinico-pathological variables were collected for analyses including sex, age at diagnosis, tumor type according to the WHO classification, size, stage, relapse date and last news date. Samples characteristics are detailed in Table 1.

**Table 1:** Thymic epithelial tumors characteristics

	*N* = 103 patients
Histology WHO 2015	
Thymoma B2/B3	18 (17%)
Thymoma B3	35 (34%)
Thymoma B3/C	4 (4%)
Thymic carcinoma	46 (45%)
Type	
Biopsy	20 (19%)
Surgical resection	83 (81%)
Tumor	
Initial resection	89 (86%)
Recurrence	4 (4%)
Metastasis	10 (10%)

### Immunohistochemistry

All antibodies were tested on whole sections of tumors instead of TMA to assess staining heterogeneity.

#### PD-L1 antibodies

A first set of three antibodies was tested: Clone E1L3N (Cell Signaling Technology, Danvers, MA, USA), clone 22C3 (Pharm Dx kit, DAKO, Agilent Technology, Santa Clara, CA, USA) and clone SP142 (Spring Bioscience, Pleasanton, CA, USA). We completed the study with the SP263 assay (Ventana Medical System, Tucson, USA) when it became commercially available, but we could only determine the SP263 status for 83 samples. SP142 was tested both as CA on Benchmark Ultra or as LDT on Dako autostainer, E1L3N was tested as LDT on Dako autostainer. PD-L1 22C3 PharmDx assay was performed on Dako Autostainer 48 according to the manufacturer’s instructions. PD-L1 SP263 commercial assay was performed on Benchmark Ultra according to the manufacturer’s instructions. See Table 2 for further details.

**Table 2:** Antibodies and technical data

Antibody	Clone	Provider	Visualisation system	Dilution
PD-L1	E1L3N (LDT)	Cell Signaling Technology	Envision Flex Sytem Dako	1/500
PD-L1	22C3 (CA)	Agilent (Dako)	Envision Flex Sytem Dako	Prediluted
PD-L1	SP263 (CA)	Roche Ventana	Optiview system Ventana	Prediluted
PD-L1	SP142 (LDT)	Roche (Spring biosciences)	Envision Flex Sytem Dako	1/100
PD-L1	SP142 (CA)	Roche (Spring biosciences)	Optiview system Ventana	1/60
PD1	Nat105	Roche Ventana	Optiview system Ventana	Prediluted
CD8	SP57	Roche Ventana	Optiview system Ventana	Prediluted
PD-L2	D7U8C	Cell Signaling Technology	Envision Flex Sytem Dako	1/100

#### Other antibodies

Tested were: PD1 (NAT105, Ventana Medical System, Tucson, USA), CD8 (SP57, Ventana Medical System, Tucson, USA), PD-L2 (D7U8C, Cell Signaling Technology, Danvers, MA, USA).

Sections were deparaffinized, rehydrated and heated for antigen retrieval, 20 min in high buffer (DAKO) (PD-L1 SP142, PD-L1 E1L3N, and PD-L2), and 64 min CC1 (PD1 and CD8). Immunohistochemistry was performed on Dako Link autostain (PD-L1 and PD-L2) with envision flex system or on Ventana Benchmark (PD1 and CD8) with Optiview revelation system. Slides were incubated with primary antibodies 1 h at a 1/100 dilution (PD-L1, SP-142), 1 h at a 1/500 dilution (PD-L1, E1L3N), 1 h at 1/100 dilution (PD-L2), 32 min (prediluted PD1) and 20 min (prediluted CD8).

##### Technical data are summarized in Table 2.

All the slides were incubated in di-amino-benzindine (DAB) and counterstained with hematoxylin, dehydrated and mounted. Two independent experienced readers examined the slides and evaluated the percentage and intensity of epithelial and immune stained cells.

**Fig. 1:**
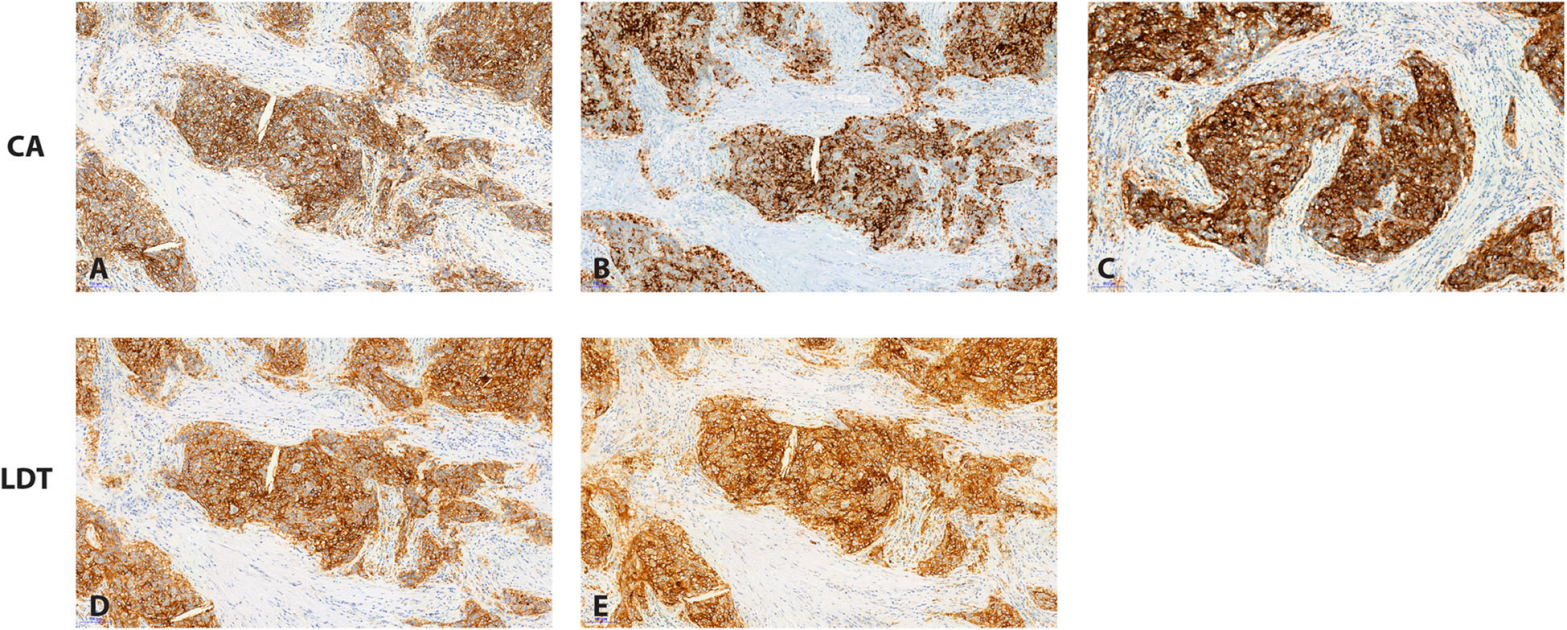
PD-L1 Thymoma staining comparaison. Commercial Assays (CA): PDL1 22C3 PharmDx Dako (**a**); Ventana PD-L1 SP142 Assay (**b**), Ventana PD-L1 S263 Assay (**c**) Laboratory developed test (LDT): PD-L 1-E1L3N cell signaling technology (**d**); PDL1-SP142 Ventana (**e**)

PD-L1 positive epithelial cells were defined as having a clear peripheral membrane staining according to the scoring already used in lung cancer clinical trials. Cytoplasmic staining was not considered as positive (Fig. 1).

In order to evaluate the role of staining intensity, a semi-quantitative scoring was used with three levels of intensity 1+, 2+ and 3+ and a H score was established as previously described in the literature. For the immune cells we evaluated both intensity and percentage.

### Statistical data analysis

Data were summarized by frequency and percentage for categorical variables and by median and range for continuous variables.

Comparisons between antibodies were performed using the Wilcoxon signed rank test for paired comparisons. Correlations were assessed using the Spearman’s rank correlation coefficient.

IHC expressions were then dichotomized according to thresholds 1 and 50% (negative vs. positive). Concordance was evaluated using Kappa Statistics. The association between IHC level (positive vs. negative) and clinical covariates were performed with Pearson’s chi-squared or Fisher’s exact tests.

Patients with only metastatic samples were excluded of the survival analysis study. All survival times were calculated from the collection date of samples (initial diagnosis) and estimated by the Kaplan Meier method with 95% confidence intervals (CI), through the use of the following first-event definitions: progression or death for Relapse Free Survival (RFS) and death for Overall Survival (OS). Patients alive were censored at the date of last follow-up. Univariable analyses were performed using the log-rank test. All reported *p*-values were two-sided. For all statistical tests, differences were considered significant at a 5% level. All statistical analyses were conducted using STATA 12.0 software.

## Results

### Patients’ characteristics

We analyzed 103 patients including 53 TB3 and 50 TC. Samples were issued from surgical specimens (*n* = 83) or biopsy (*n* = 20). Most of patients were men with a median age of 57 years old. Patients’ characteristics are detailed in Table 3.

**Table 3:** Patients characteristics

	*N* = 103 patients
Sex	
Male	62 (60%)
Female	41 (40%)
Paraneoplasic syndrome	
No	32 (76%)
Yes	10 (24%)
Missing	61
Recurrence	
No	14 (22%)
Yes	50 (78%)
Missing	39
Survival	
Alive	72 (81%)
Dead	17 (19%)
Missing	14 (non primary tumors)

### PD-L1 immunostaining

**Fig. 2:**
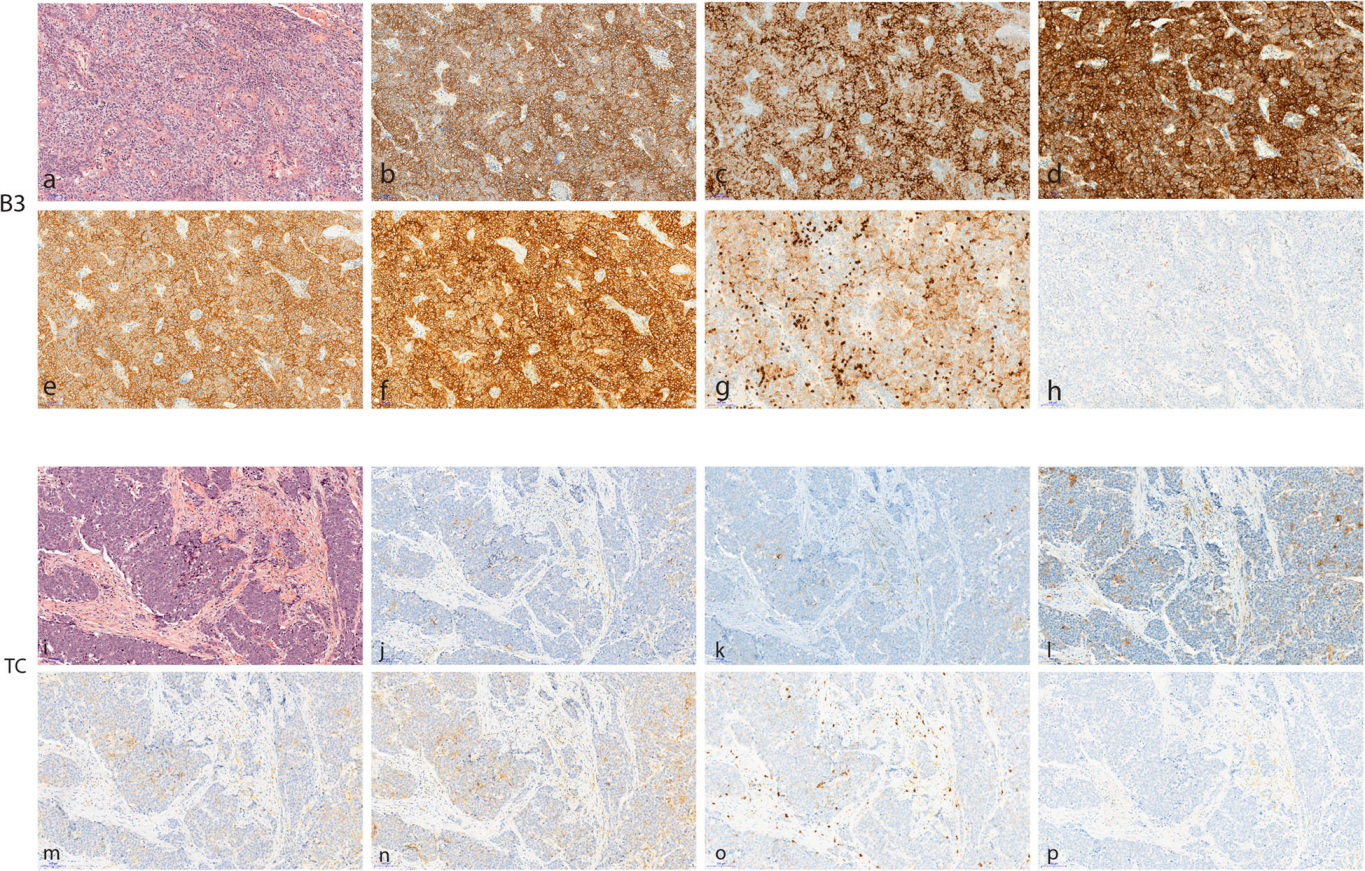
Comparison of B3 Thymomas (TB3) (**a** to **h**) and Thymic Carcinoma (TC) (**I** to **p**) staining with Commercial Assays (CA) and Laboratory developed tests (LDT). HE staining (**a**, **i**); CA, PD-L1 22C3 PharmDx Dako (**b**, **j**); CA, Ventana PDL1 SP142 Assay (**c**, **k**); CA, Ventana PD-L1 S263 Assay (**d**, **l**); LDT PD-L1-E1L3N cell signaling technology (**e**, **m**); LDT PD-L1-SP142 Ventana (**f**, **n**); CA CD8-SP57 (**g**, **o**); CA PD1-NAT105 (**h**,**p**)

We have first analyzed PDL1 expression using four different antibodies (Table 2). PD-L1 expression was found positive using a 50% threshold in approximatively half of the patients with reproducible results across the antibodies: 51% with 22C3 pharm DX assay, 52% with E1L3N antibody on Dako Autostainer, 51% with SP142 antibody on Dako Autostainer, 53% with SP263 CA on Ventana Benchmark Ultra. The only exception was observed with SP142 antibody used with Ventana Benchmark Ultra which was positive in only 20% of the analyzed tumors (Fig. 2). Using a 1% threshold, around 80% of the tumors were positive with all antibodies except for SP142 on Ventana automate (64%) (Table 4).

**Table 4:** Percentage of positivity of tumor cells with 50 and 1% threshold

Positive tumor cells 50% threshold	TB3	TC	*P* value	TB3 and TC
PD-L1 E1L3N (LDT)	44 (83%)	10 (20%)	< 0,0001	54/103 (52%)
PD-L1 22C3 (CA)	41 (77%)	12 (24%)	< 0,0001	53/103 (51%)
PD-L1 SP142 on Dako (LDT)	42 (81%)	10 (20%)	< 0,0001	52/102 (51%)
PD-L1 SP142 on ventana (CA)	4 (29%)	1 (09%)	=0,3406	5/25 (20%)
PD-L1 Sp263 (CA)	45 (92%)	10 (23%)	< 0,0001	55/93 (53%)
Positive tumor cells 1% threshold	TB3	TC	*P* value	TB3 and TC
PD-L1 E1L3N (LDT)	51 (96%)	36 (72%)	=0,0007	87/103 (84%)
PD-L1 22C3 (CA)	49 (92%)	35 (70%)	=0,0033	84/103 (82%)
PD-L1 SP142 on Dako (LDT)	48 (92%)	33 (66%)	=0,0010	81/102 (79%)
PD-L1 SP142 on ventana (CA)	10 (71%)	6 (54%)	=0,4341	16/25 (64%)
PD-L1 Sp263 (CA)	48 (98%)	32 (73%)	=0,0005	80/93 (86%)

PD-L1 expression on epithelial cells was higher and more diffuse in TB3 compared to TC for all antibodies (*p* < 0.0001) ranging from 81 to 92% for TB3 and 20 to 24% for TC with 50% cut-off and from 92 to 98% for TB3 and 66 to 73% for TC using 1% cutoff (Table 4). We found a significant difference for both 1 and 50% cut-off with all antibodies except SP142 on Ventana automate (Table 4).

As in most studies published concerning other tumors, the staining intensity and the H-score did not appear to be relevant.

PD-L1 expression was not associated with tumor stage no matter the antibody applied. Interestingly, PD-L1 was statistically more frequently expressed in tumors with paraneoplastic syndrome regardless the antibodies. On the contrary, sex, age and tumor stage had no impact on PD-L1 expression.

### Concordance between PD-L1 antibodies on epithelial tumors cells

We next analyzed the correlation between the antibodies used. Therefore, a good concordance was observed between the four antibodies on TC and TB3 using both 1 and 50% cut-off (Table 5 and 6).

**Table 5:**
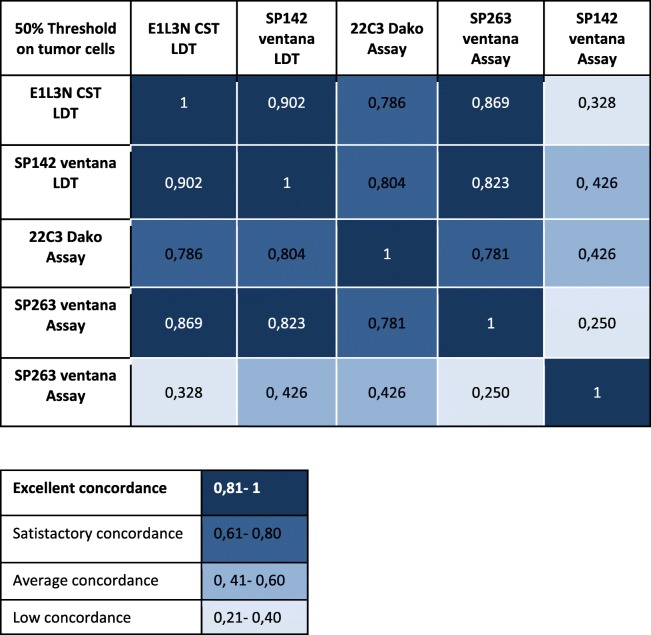
Concordance between PD-L1 antibodies on tumors cells 50% threshold

**Table 6:**
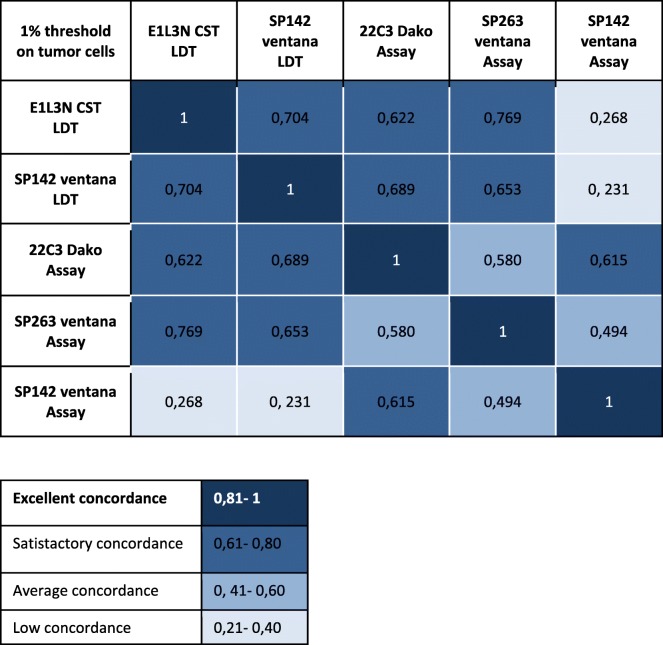
Concordance between PD-L1 antibodies on tumors cells 1% threshold

Interestingly, we obtained a similar staining for all antibodies tested as LDT (Table 2) and for SP263 Assay. SP142 is less expressed when tested as CA on the Benchmark Ultra: this assay has been considered in concordance studies published in the field of lung cancer as less relevant than the others.

Concerning immune cells, we found a low concordance between antibodies and a significant difference between TB3 and TC was found only using E1L3N antibody (Table 7).

**Table 7:** Percentage of positivity of lymphoid cells with 50 and 1% threshold

Positive lymphoid cells 50% threshold	TB3	TC	*P* value
PD-L1 E1L3N (LDT)	0/53 (0%)	10/50 (20%)	=0,0004
PD-L1 22C3 (CA)	0/53 (0%)	1/50 (2%)	=0,4854
PD-L1 SP142 on Dako (LDT)	0/51 (0%)	3/50 (6%)	=0,1171
PD-L1 SP142 on ventana (CA)	0/14 (0%)	0/11 (0%)	
PD-L1 Sp263 (CA)	0/46 (0%)	0/42 (0%)	
Positive lymphoid cells 1% threshold	TB3	TC	*P* value
PD-L1 E1L3N (LDT)	23/53 (43%)	24/53 (48%)	=0,6392
PD-L1 22C3 (CA)	16/53 (30%)	25/50 (50%)	=0,0401
PD-L1 SP142 on Dako (LDT)	12/51 (23%)	18/50 (36%)	=0,1703
PD-L1 SP142 on ventana (CA)	4/14 (27%)	6/11 (54%)	=0,2406
PD-L1 Sp263 (CA)	5/46 (11%)	18/42 (43%)	=0,0006

### Other biomarkers analysis

Concerning other biomarkers and clinical correlations, we found no expression for PDL2. Concerning CD8, all the samples presented an immune cells staining. Using a 1% threshold, we found no significant difference between TB3 and TC. Conversely, using a 50% threshold we found a less frequent expression in TC (*p* < 0,0001). PD1 stained no tumor cell, and for immune cells in most of cases the proportion of positive cells was around 1% without any significant difference between TB3 and TC. We found no correlation between PD1 and CD8 expression by immune cells and PD-L1 expression on tumor cells (Table 8).

**Table 8:** CD8 and PD1 positivity in tumor and lymphoid cells

Positive cells	TB3 *N* = 53	TC *N* = 50	*P* value	TB3 and TC Positive cells
CD8 tumor cells	2 (4%)	1 (2%)	= 1000	3/103 (3%)
CD8 lymphoid cells	48 (91%)	23 (46%)	< 0,0001	71/103 (69%)
PD1 tumor cells	0 (0%)	0 (0%)	–	0 (0%)
PD1 lymphoid cells	0 (0%)	0 (0%)	–	0 (0%)

### Prognostic value of PD-L1 and other biomarkers

**Fig. 3:**
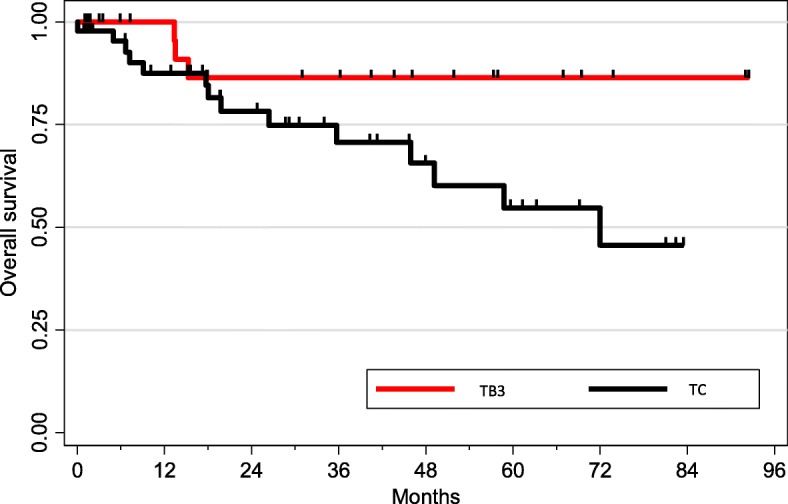
Overall survival according to histology

**Fig. 4:**
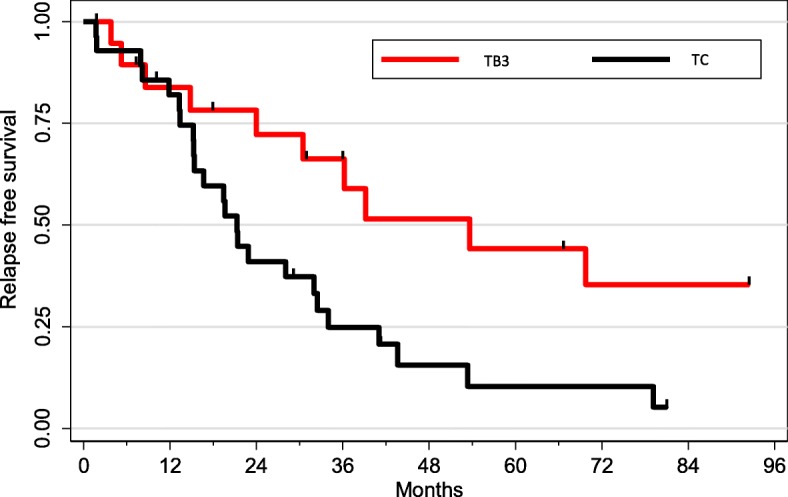
Relapse free survival according to histology

**Fig. 5:**
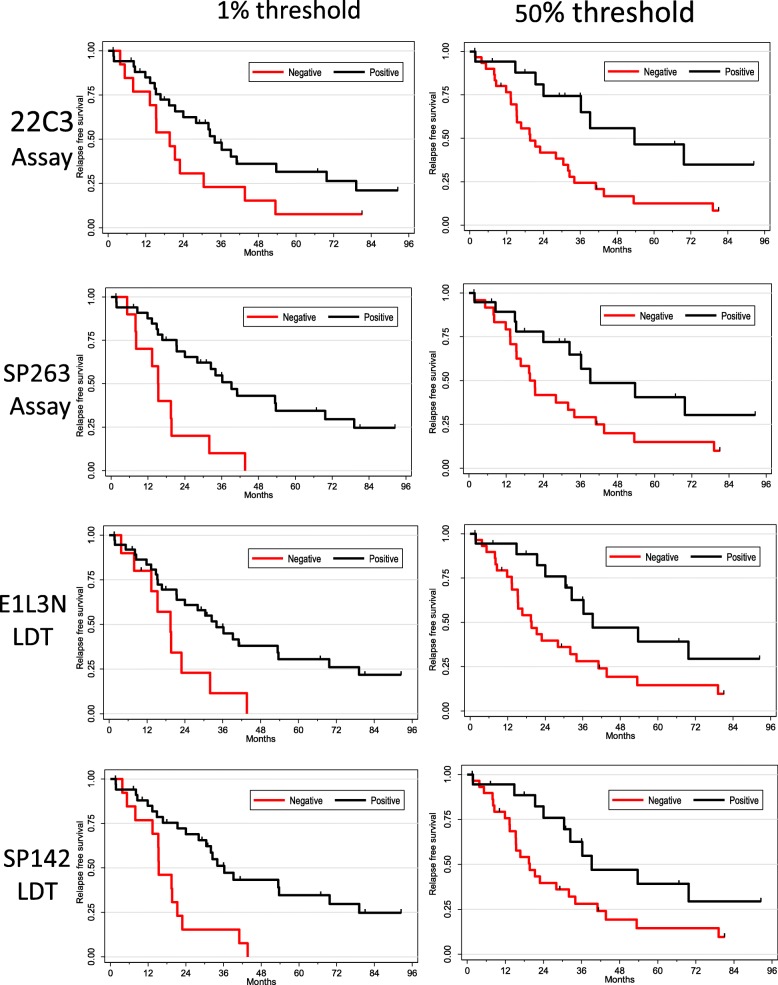
Relapse free survival according to antibody and 50% or 1% threshold

Median follow-up was 41 months (data available for 89 patients). One-year, three-year and five-year survival were 92, 77 and 67% respectively. As expected, survival was superior in TB3 when compared to TC (*p* = 0.04, Fig. 3). There was no correlation between PD-L1 expression and overall survival in the whole population. Progression free survival available for 48 patients, was 82, 55 and 40% at one, two and 3 years respectively and was also worse in TC compared to TB3 (*p* = 0.01) (Fig. 4). We then analyzed the impact of PD-L1 expression on PFS patients. In the subgroup of TB3, PD-L1 expression was significantly associated with a better PFS no matter which antibody was used (Fig. 5). PFS was almost double in patients with PDL1 expression regardless the antibody used for PD-L1 detection and the cut-off (1% vs. 50%).

## Discussion

We here demonstrated that B3 thymoma and thymic carcinoma frequently express PD-L1. Our study strengths are its large size, the use of four different clones of PD-L1 antibodies and the context of a national cohort with well-annotated tumors and validated diagnosis by a panel of expert pathologists. Our research was restricted to B3 thymoma and thymic carcinoma because immunotherapy use is limited to these subtypes and because their epithelial component fits for epithelial expression analysis of PD-L1.

PDL1 expression in thymic tumors has been recently reported with conflicting data regarding its prognostic value. Katsuya et al. performed a tissue microarray (TMA) of thymomas and thymic carcinomas with a rabbit monoclonal PD-L1 antibody (clone E1L3N) [13]. They have reported that PD-L1 expression increased according to tumor types (from 23% in types A, AB and B) vs 70% in thymic carcinoma. PD-L1 was not related to prognostic. Padda et al. also performed a TMA analysis with two clones (5H1 and 15) [11]. PD-L1 high scores were more frequent in TETs than in thymic controls (68.1% versus 17.6%). PD-L1 scores and histology were significantly correlated, with higher intensity staining in B1 and B2 thymomas. PD-L1 expression was associated with a significantly worse overall survival. Yokohama et al. performed a monocentric study using a rabbit monoclonal anti-PD-L1 (EPR1161 Abcam) and reported that 54% of thymomas revealed high PD-L1 expression [12]*.* In a recent study, PD-L1 was found positive in 61/100 cases (61%) including 14/26 thymic carcinomas (54%) and 47/74 thymomas (64%)*.* There was no statistical difference between PD-1 or PD-L1 expression status and other clinicopathological parameters including overall survival [16]. In addition, in another cohort, PD-L1 was expressed in 90% of non-neoplastic thymus, 92% of thymomas, and 50% of carcinomas tissues, with significantly higher scores in B2 and B3 thymomas and carcinomas than in AB and B1 thymomas [17]. In a more recent work on 35 resected thymoma, PD-L1 expression was detected in 83% (29/35) tumor samples, including 100% (3/3) of thymic carcinoma patients and 81% (26/32) of thymoma patients using 22C3 antibody [18]. None of these papers have compared companion tests of lung clinical trials. Conflicting data have been recently published regarding the prognostic value of PDL1. Wei et al. found no impact of PD-L1 expression on survival but high PD-L1 was associated with advanced Masaoka staging and high-grade histology in surgically treated thymoma [19]*).* In two other studies, PD-L1 expression had no impact of PFS and 5 yr survival [18, 20]*.* In patients with advanced thymic carcinoma, the median PFS was higher in the low PD-L1 group vs the high PD-L1 group (23.5 vs 13.3 months) [21]. Lastly Arbour et al. reported that PD-L1 expression was more common in thymomas compared to thymic carcinoma and was associated with longer overall survival (*Arbour KC, PLoS One 2017*) in line with our findings.

This is the first study that compares four different clones of PD-L1 antibodies, three of them previously used in lung or melanoma clinical trials, the fourth one E1L3N being used in numerous clinical studies. Our work allowed us to develop reproducible and comparable immunohistochemical processes for the four antibodies tested: 22C3 pharmDX and SP263 assays are captive tests and did not necessitate any technical adaptation. The SP142 has been recently developed to become a captive test too. However, in our study, we have tested the free antibody on two different automates and we found a high concordance with the other antibodies concerning the epithelial cells staining only when it was used on Dako autostainer 48. The last one, E13LN, required a relatively easy technical adaptation. Therefore, we have demonstrated that after some technical adaptations, the four clones may provide very reproducible results. The preanalytic phase has been shown to be critical and to have a real impact on PD-L1 expression. We have here restrained our study to formalin-fixed tissues but there was a great heterogeneity in our series with tumors arising from different centers and some old archived cases. However, we have obtained homogeneous results that favor the hypothesis of relatively robust antibodies. These results are similar to those reported in lung cancer KEYNOTE 010 trial that had shown a good reproducibility of results between archived tissues and fresh biopsies [22].

Our work has shown a high reproducibility between the four clones for the epithelial cells staining which is usually clear-cut. Immune cells staining is less clear, sometimes granular and seems to be more frequent in thymic carcinoma and tends to be inversely expressed than in epithelial cells. This may be due to the particular morphology of thymic tumors: in B3 thymomas there are very few immune cells whereas, in thymic carcinoma they are usually well separated from the epithelial cells without interface patterns. The difference in PDL1 epithelial tumor expression is clear between B3 thymomas, which usually show a high and diffuse expression, and thymic carcinomas, which seem to have a more focal and heterogeneous expression. In our series, the 1 and 50% positive tumor epithelial cells thresholds appear to be highly significant in order to differentiate B3 thymomas from thymic carcinomas. These thresholds have been reported to be reliable to a good clinical response to pembrolizumab treatment in Lung cancer trials [23]. The 50% threshold is now considered for the first line treatment use of pembrolizumab in lung cancer and the 1% threshold for its second line use.

Regarding immune cells we found no significant threshold but interestingly we came up with a significant difference between TB3 and TC only for E1L3N clone.

Noteworthily, other immune biomarkers may be of interest in thymoma. The frequency of MSI has been reported around 10% in a series of 55 patients [24]. High tumor mutational burden was observed frequently in thymic carcinoma and was associated with worse survival [25]. No specific immune-related signature was reported in genetic characterization of thymoma [26].

Thymic tumours management is a paradigm of cooperation between clinicians, surgeons, and pathologists from establishing the diagnosis to organizing the therapeutic strategy. The PD1-PD-L1 axis can be targeted thanks to immune checkpoint inhibitors with clinical success observed across many tumor types including thoracic malignancies. Given the high frequency of PD-L1 expression in our series we anticipated that it may be a promising target in thymomas. Preliminary results of a recent phase II trial have reported interesting activity of pembrolizumab in this disease. Conversely attention should be paid on the risk of immune-related side effects in a disease that is known for the frequency of paraneoplastic syndrome.

Based on our results, patients with stage B3 thymoma appears to be the best candidates for such a strategy because of the high expression of PD-L1, but some thymic carcinomas with PD-L1 expression on epithelial or even immune cells may also be concerned.

Immunotherapy is currently not a standard-of-care in thymic epithelial tumors and should even not be delivered in an off-label setting, especially if the patients are eligible for ongoing clinical trials. Preliminary results from clinical trials have been recently reported. In a Korean study, treatment of TET with pembrolizumab was associated with 2 responses out of seven thymoma and 5 out of 26 thymic carcinoma, with 6.1 months median progression-free survival for both groups [27]. An American study also with pembrolizumab have reported a Response Rate at 22.5% on 40 patients. A high incidence of immune-related side effect was also found [28].. In Europe, the European Organization for Research and Treatment of Cancer (EORTC) and the European Thoracic Oncology Platform (ETOP) are now starting a single-arm, multicentre, phase II study - the NIVOTHYM trial - to assess the efficacy of nivolumab alone or combined with ipilimumab in patients with advanced, refractory type B3 thymomas and thymic carcinomas (NCT03134118).

## Conclusion

We demonstrated the frequency of PD-L1 expression in B3 thymoma and, to a lesser extent, of thymic carcinoma. PD-L1 expression analysis can be performed with commercially available antibodies otherwise validated with robust and reproducible results. Our findings pave the way for the personalized use of PD1-PD-L1 inhibitors in these tumors.

## Abbreviations

CD8: Cluster differentiation 8
DAB: Di-Amino-Benzindine
FFPE: Formalin-Fixed Paraffin Embedded
IHC: Immuno Histo Chemistry
LDT: Laboratory Developed Test
PD1: Programmed cell death 1
PD-L1: Programmed Death Ligand 1
PD-L2: Programmed Death Ligand 1
TB3: B3 Thymoma
TC: Thymic Carcinomas
TET: Thymic Epithelial Tumor
TMA: Tissue MicroArray

## References

[1] Girard N (2014). Thymic tumors: adopting an orphan thoracic tumor as a model of personalized medicine. J Thorac Oncol.

[2] Marx A, Strobel P, Badve SS, Chalabreysse L, Chan JK, Chen G (2014). ITMIG consensus statement on the use of the WHO histological classification of thymoma and thymic carcinoma: refined definitions, histological criteria, and reporting. J Thorac Oncol.

[3] Girard N (2012). Chemotherapy and targeted agents for thymic malignancies. Expert Rev Anticancer Ther.

[4] Remon J, Girard N, Mazieres J, Dansin E, Pichon E, Greillier L (2016). Sunitinib in patients with advanced thymic malignancies: cohort from the French RYTHMIC network. Lung Cancer.

[5] Giaccone G, Rajan A, Ruijter R, Smit E, van Groeningen C, Hogendoorn PC (2009). Imatinib mesylate in patients with WHO B3 thymomas and thymic carcinomas. J Thorac Oncol.

[6] Wheler J, Hong D, Swisher SG, Falchook G, Tsimberidou AM, Helgason T (2013). Thymoma patients treated in a phase I clinic at MD Anderson Cancer Center: responses to mTOR inhibitors and molecular analyses. Oncotarget.

[7] Guibert N, Delaunay M, Mazieres J (2015). Targeting the immune system to treat lung cancer: rationale and clinical experience. Ther Adv Respir Dis.

[8] Fehrenbacher L, Spira A, Ballinger M, Kowanetz M, Vansteenkiste J, Mazieres J (2016). Atezolizumab versus docetaxel for patients with previously treated non-small-cell lung cancer (POPLAR): a multicentre, open-label, phase 2 randomised controlled trial. Lancet.

[9] Borghaei H, Paz-Ares L, Horn L, Spigel DR, Steins M, Ready NE (2015). Nivolumab versus Docetaxel in advanced nonsquamous non-small-cell lung Cancer. N Engl J Med.

[10] Giaccone G, Kim C, Thompson J, McGuire C, Kallakury B, Chahine JJ (2018). Pembrolizumab in patients with thymic carcinoma: a single-arm, single-Centre, phase 2 study. Lancet Oncol.

[11] Padda SK, Riess JW, Schwartz EJ, Tian L, Kohrt HE, Neal JW (2015). Diffuse high intensity PD-L1 staining in thymic epithelial tumors. J Thorac Oncol.

[12] Yokoyama S, Miyoshi H, Nishi T, Hashiguchi T, Mitsuoka M, Takamori S (2016). Clinicopathologic and prognostic implications of programmed death ligand 1 expression in Thymoma. Ann Thorac Surg.

[13] Katsuya Y, Fujita Y, Horinouchi H, Ohe Y, Watanabe S, Tsuta K (2015). Immunohistochemical status of PD-L1 in thymoma and thymic carcinoma. Lung Cancer.

[14] Chalabreysse L (2014). Thomas De Montpreville V, De Muret a, Hofman V, Lantuejoul S, Parrens M, et al. [Rythmic-pathology: the French national pathology network for thymic epithelial tumours]. Ann Pathol.

[15] Marx A, Chan JK, Coindre JM, Detterbeck F, Girard N, Harris NL (2015). The 2015 World Health Organization classification of tumors of the Thymus: continuity and changes. J Thorac Oncol.

[16] Weissferdt A, Fujimoto J, Kalhor N, Rodriguez J, Bassett R, Wistuba II (2017). Expression of PD-1 and PD-L1 in thymic epithelial neoplasms. Mod Pathol.

[17] Marchevsky AM, Walts AE (2017). PD-L1, PD-1, CD4, and CD8 expression in neoplastic and nonneoplastic thymus. Hum Pathol.

[18] Owen D, Chu B, Lehman AM, Annamalai L, Yearley JH, Shilo K (2018). Expression patterns, prognostic value, and Intratumoral heterogeneity of PD-L1 and PD-1 in Thymoma and Thymic carcinoma. J Thorac Oncol.

[19] Wei YF, Chu CY, Chang CC, Lin SH, Su WC, Tseng YL (2018). Different pattern of PD-L1, IDO, and FOXP3 Tregs expression with survival in thymoma and thymic carcinoma. Lung Cancer.

[20] Hakiri S, Fukui T, Mori S, Kawaguchi K, Nakamura S, Ozeki N (2019). Clinicopathologic features of Thymoma with the expression of programmed death ligand 1. Ann Thorac Surg.

[21] Duan J, Liu X, Chen H, Sun Y, Liu Y, Bai H (2018). Impact of PD-L1, transforming growth factor-beta expression and tumor-infiltrating CD8(+) T cells on clinical outcome of patients with advanced thymic epithelial tumors. Thorac Cancer.

[22] Herbst RS, Baas P, Kim DW, Felip E, Perez-Gracia JL, Han JY (2016). Pembrolizumab versus docetaxel for previously treated, PD-L1-positive, advanced non-small-cell lung cancer (KEYNOTE-010): a randomised controlled trial. Lancet.

[23] Garon EB, Rizvi NA, Hui R, Leighl N, Balmanoukian AS, Eder JP (2015). Pembrolizumab for the treatment of non-small-cell lung cancer. N Engl J Med.

[24] Inoue M, Starostik P, Zettl A, Strobel P, Schwarz S, Scaravilli F (2003). Correlating genetic aberrations with World Health Organization-defined histology and stage across the spectrum of thymomas. Cancer Res.

[25] Wang X, Li M (2019). Correlate tumor mutation burden with immune signatures in human cancers. BMC Immunol.

[26] Yu L, Ke J, Du X, Yu Z, Gao D (2019). Genetic characterization of thymoma. Sci Rep.

[27] Cho J, Kim HS, Ku BM, Choi YL, Cristescu R, Han J (2019). Pembrolizumab for patients with refractory or relapsed Thymic epithelial tumor: an open-label phase II trial. J Clin Oncol.

[28] Giaccone G, Thompson J, McGuire C, Manning M, Kallakury B, Chahine JJ (2017). Pembrolizumab in patients with recurrent thymic carcinoma: Results of a phase II study. J Clin Oncol.

